# Functional significance of co-occurring mutations in *PIK3CA* and *MAP3K1* in breast cancer

**DOI:** 10.18632/oncotarget.25118

**Published:** 2018-04-20

**Authors:** Alvaro Avivar-Valderas, Robert McEwen, Amir Taheri-Ghahfarokhi, Larissa S. Carnevalli, Elizabeth L. Hardaker, Marcello Maresca, Kevin Hudson, Elizabeth A. Harrington, Francisco Cruzalegui

**Affiliations:** ^1^ Translational Science, Oncology, IMED Biotech Unit, AstraZeneca, Cambridge, UK; ^2^ Translational Genomics, Discovery Sciences, IMED Biotech Unit, AstraZeneca, Gothenburg, Sweden; ^3^ Bioscience, Oncology, IMED Biotech Unit, AstraZeneca, Cambridge, UK; ^4^ Current address: TiGenix, Parque Tecnológico de Madrid, Tres Cantos, Madrid, Spain; ^5^ Current address: 2TheNth, Adelphi Mill, Bollington, Macclesfield, UK; ^6^ Current address: Pierre Fabre R&D Centre, Toulouse, France

**Keywords:** luminal A, breast cancer, *PIK3CA*, *MAP3K1*, CRISPR gene editing

## Abstract

The PI3Kα signaling pathway is frequently hyper-activated in breast cancer (BrCa), as a result of mutations/amplifications in oncogenes (e.g. *HER2*), decreased function in tumor suppressors (e.g. *PTEN*) or activating mutations in key components of the pathway. In particular, activating mutations of *PIK3CA* (~45%) are frequently found in luminal A BrCa samples. Genomic studies have uncovered inactivating mutations in *MAP3K1* (13-20%) and *MAP2K4* (~8%), two upstream kinases of the JNK apoptotic pathway in luminal A BrCa samples. Further, simultaneous mutation of *PIK3CA* and *MAP3K1* are found in ~11% of mutant *PIK3CA* tumors. How these two alterations may cooperate to elicit tumorigenesis and impact the sensitivity to PI3K and AKT inhibitors is currently unknown. Using CRISPR gene editing we have genetically disrupted *MAP3K1* expression in mutant *PIK3CA* cell lines to specifically create *in vitro* models reflecting the mutational status of *PIK3CA* and *MAP3K1* in BrCa patients. *MAP3K1* deficient cell lines exhibited ~2.4-fold increased proliferation rate and decreased sensitivity to PI3Kα/δ(AZD8835) and AKT (AZD5363) inhibitors (~2.61 and ~5.23-fold IC_50_ increases, respectively) compared with parental control cell lines. In addition, mechanistic analysis revealed that *MAP3K1* disruption enhances AKT phosphorylation and downstream signaling and reduces sensitivity to AZD5363-mediated pathway inhibition. This appears to be a consequence of deficient MAP3K1-JNK signaling increasing IRS1 stability and therefore promoting IRS1 binding to p85, resulting in enhanced PI3Kα activity. Using 3D-MCF10A-PI3Kα^H1047R^ models, we found that MAP3K1 depletion increased overall acinar volume and counteracted AZD5363-mediated reduction of acinar growth due to enhanced proliferation and reduced apoptosis. Furthermore, *in vivo* efficacy studies revealed that MAP3K1-deficient MCF7 tumors were less sensitive to AKT inhibitor treatment, compared with parental MCF7 tumors. Our study provides mechanistic and *in vivo* evidence indicating a role for *MAP3K1* as a tumor suppressor gene at least in the context of *PIK3CA*-mutant backgrounds. Further, our work predicts that *MAP3K1* mutational status may be considered as a predictive biomarker for efficacy in PI3K pathway inhibitor trials.

## INTRODUCTION

BrCa is characterized by its heterogeneity [1, 2]. Traditionally classified according to histological criteria, BrCa has also been categorized according to gene expression and recently according to genomic alterations. Large-scale DNA sequencing [3–5] has provided a mutational landscape of BrCa. One of the most prevalent genes mutated in cancer is *PIK3CA* which is mutated in 45% of luminal A BrCa cases [3]. Multiple drug-discovery organizations have been developing PI3Kα inhibitors for many years but response rates are low and no drug against this target has yet been approved. We hypothesized that perhaps response rates are low because other mediators that are frequently mutated in conjunction with *PIK3CA* may counteract the effect of PI3Kα inhibitors and other PI3K pathway inhibitors, promoting resistance. Thus, we interrogated published genomic databases to identify genes that were frequently mutated in BrCa patients with mutant *PIK3CA* (*mPIK3CA*). In addition to genes previously reported to have a role in BrCa some novel genes were identified in those studies [3]. Because of the frequency of co-occurrence with *mPIK3CA* we were particularly interested in *MAP3K1* and *MAP2K4*, two upstream kinases of the JNK apoptotic pathway. In particular, mutations in *MAP3K1* are found in 13–20% of luminal A BrCa. The majority of these are loss of function (LoF) mutations suggesting a tumor suppressor role for this kinase [3, 5]. However despite this genomic evidence, the role of *MAP3K1* in tumorigenesis is not completely clear, since while several studies support the notion of *MAP3K1* having a tumor suppressor role [4, 6–9], others have reported that *MAP3K1* fuels tumor progression and metastasis [9–11].

MAP3K1 or MEKK1 (MEK kinase 1) is a serine-threonine kinase of 196 kDa [12] belonging to the mitogen-activated protein kinase kinase kinase 1 (MAP3K) family [12, 13]. MAP3K1 is activated by a variety of stimuli (e.g. growth factors, cytokines) and cell stresses (e.g. loss of adhesion, microtubule disruption, cold temperature). Of the 19 members of the MAP3K family, MAP3K1 is the only member of the group which contains specific domains (PHD, SWIN and RING motifs) and features (caspase cleavage site and E3 ligase activity) [7, 14, 15]. Caspase-3 targeting of MAP3K1 produces a 91 kDa c-terminal catalytic fragment that localizes in the cytoplasm and has selectivity toward JNK but not to ERK1/2 and is thought to play a role in the induction of apoptosis [16]. By virtue of transducing pro-apoptotic and pro-survival signaling via caspase-3 and ERK/NF-κB [17, 18], respectively, MAP3K1 appears to play a critical role in balancing cell fate decisions [13, 19, 20].

Currently, there is limited understanding of the cross-talk between PI3Kα and MAP3K1 signal transduction pathways. AKT has been reported to suppress stress-induced activation of the MAP3K1-MAP2K1-JNK axis preventing apoptosis induction [21]. Hyperactivation of AKT inhibits JNK activity but not MAP3K1, suggesting that cross-talk happens at the level of MAP2K1, and indeed it was demonstrated that AKT is able to bind and phosphorylate MAP2K1 [21]. In addition, several lines of evidence suggest that JNK can cross-talk with the PI3K pathway [22–24], for example *via* insulin receptor substrate-1 (IRS1). Serine phosphorylation of IRS1 by JNK inhibits its activity by promoting IRS1 degradation *via* the proteasome; such phosphorylation can also be transduced by mTOR downstream target p70 [25, 26].

In this study we aimed to identify proteins with a relevant role in the cross-talk of PI3K and MAP3K1 pathways. The high rate of MAP3K1 LoF mutations in ER-positive BrCa patients (frequently *mPIK3CA*) suggest that pathway cross-talk might be important in tumorigenesis or response to anti-cancer therapeutics. Our work aimed to characterize whether LoF mutations in MAP3K1 provide selective advantage for tumor progression and how this impacts on resistance to PI3K pathway targeted therapies.

## RESULTS

### MAP3K1 depletion activates the PI3K pathway and reduces sensitivity to PI3K pathway inhibitors

In an attempt to understand the relationship between *PIK3CA* and *MAP3K1* and the clinical relevance of the mutational status of these genes we interrogated published genomic databases [3–5]. Using the TCGA database we found that *PIK3CA* and *MAP3K1* are mutated in 36% and 9% respectively in a cohort of BrCa patients (Supplementary Figure 1A). We observed a high incidence of these mutations within the luminal A subgroup [4] and noted high frequency of co-occurring *MAP3K1* and *PIK3CA* mutational events (Supplementary Figure 1A). Indeed, previous genomic analysis has predicted a positive correlation between *MAP3K1* and *PIK3CA* mutations supporting a case functional cooperation [4]. Further, with the exception of arginine-273 and isoleucine-761 frameshift mutations, found in 3 samples in TCGA and 1 in the Ellis *et al.* study [4], there were no distinct hot-spot mutation sites within the MAP3K1 domains (Supplementary Figure 1A). Instead, mutations in *MAP3K1* were distributed through the different domains of the gene.

We set out to test the hypothesis that MAP3K1 inactivation or depletion has an impact on the activity of the PI3K pathway and the functional activity of inhibitors of this pathway. However, we were confronted with a lack of established tumor cell models containing both activating *mPIK3CA* and inactivating *MAP3K1* alterations. To overcome this lack of models we decided to deplete endogenous levels of MAP3K1 in BrCa cell lines already containing *mPIK3CA*. We considered gene depletion to be a surrogate model for *MAP3K1* LoF mutations in tumors, since the majority of *MAP3K1* genomic mutations found in cancer patients are scattered frame-shifts insertions/deletions [3, 4] and large chromosomal deletions [5] (i.e. LoF mutations). Then, we used such models to assess the impact of *MAP3K1* mutations on the response to PI3K pathway inhibitors (AZD8835 [27] and AZD5363 [28, 29]).

**Figure 1:**
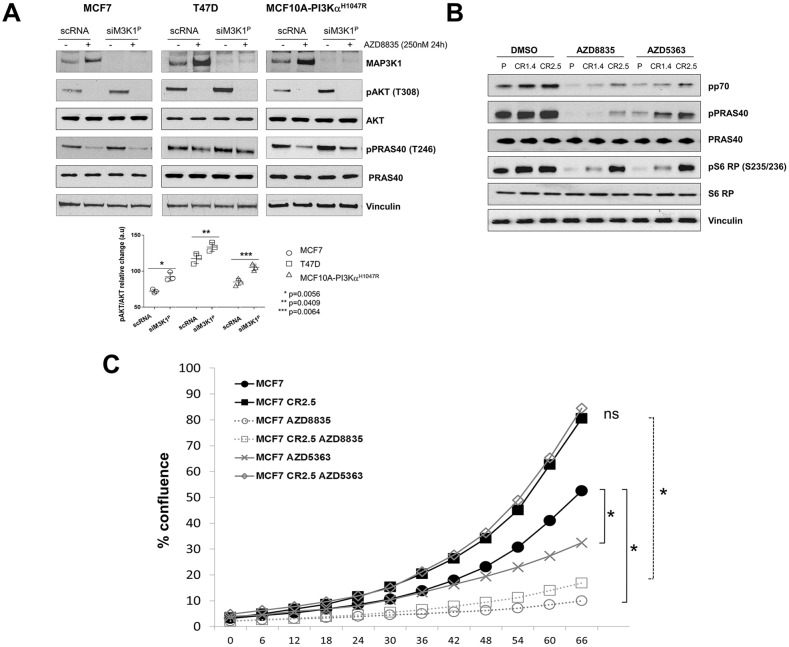
MAP3K1 inhibition reduces sensitivity to upstream PI3K pathway inhibitors **(A)** MCF7, T47D and MCF10A-PI3Kα^H1047R^ cells were cultured and transfected with *MAP3K1* (siM3K1^P^) or control (scRNA) siRNAs for 48h. Cultures were treated with 250nM AZD8835 (+) or DMSO (−) as indicated 24h prior to cell lysis. Cell lysates were then immunoblotted with indicated antibodies (Abs). P-values were determined by the Student's *t*-test. **(B)** Lysates from MCF7 parental and MAP3K1-deficient (CR1.4 and CR2.5) cells were cultured and treated with 250 nM AZD8835 or 25 nM AZD5363 as indicated. Lysates were collected and processed for immunoblotting with the indicated Abs. **(C)** IncuCyte cell proliferation assays showing relative % of confluence of MCF7 parental *versus* MAP3K1-deficient (CR1.4 and CR2.5) cultures treated with DMSO (black), AZD8835 (red) and AZD5363 (green) for 66 hours. ^*^ Statistical significance of p < 0.05, determined by the Student's *t*-test.

Firstly, using siRNA, we reduced *MAP3K1* expression in two *PIK3CA*-mutant tumor cell lines (MCF7 and T47D) and in a breast cell line (MCF10A) that had been genetically engineered to express a common *PIK3CA* (H1047R) mutant (hereafter referred to as MCF10A-PI3Kα^H1047R^). In scrambled control RNA (scRNA) treated cells, following treatment with PI3Kα/δ inhibitor AZD8835 [27] we observed a significant increase in the expression levels of MAP3K1 in all cell lines. Also, siRNA targeting of *MAP3K1* resulted in an increase in pAKT (T308) levels; consistent with this, AKT substrate, PRAS40 was also more highly phosphorylated (pT246) in such cells (Figure 1A, lower panel quantification). Furthermore, we observed that *MAP3K1* knock-down in T47D and MCF10A-PI3Kα^H1047R^ made cells less sensitive to AZD8835-mediated pathway inhibition, as assessed by pPRAS40 signal (Figure 1A). These observations are all consistent with MAP3K1–PI3K pathway crosstalk.

To further investigate the effect of MAP3K1 depletion on PI3Kα signaling, we generated MAP3K1-deficient stable cells lines derived from MCF7 cells (harboring a *PIK3CA* E545K hotspot mutation) using precise genome editing CRISPR methodology Supplementary Figure 1B). Efficient depletion of MAP3K1 was confirmed by Sanger sequencing, NGS sequencing and western blotting (Supplementary Figure 1C-1E). We implemented bio-computational analysis to predict potential off-targets (description in materials and methods) to check the specificity of the guide RNAs in potential off-target loci, so as to select cells lines with modification specific to the MAP3K1 loci (Supplementary Figure 1F). Such validated MCF7 (CR1.4 and CR.2.5) cell lines were treated with PI3Kα/δ inhibitor AZD8835 and AKT inhibitor (AKTi) AZD5363 and compared with parental MCF7 cells (Figure 1B and Supplementary Figure 2A). We observed that MAP3K1 deficiency led to reduced inhibition of the pathway by AZD8835 and AZD5363 with a more pronounced effect on AZD5363 activity, indicated by maintained pP70S6K, pPRAS40 and pS6 ribosomal protein (RP) readouts in CR1.4 and CR.2.5 compared with parent cells. To demonstrate that these effects were not restricted to AZD5363, we used a chemically different AKTi, GDC-0068 (ipatasertib) which was similarly impacted by MAP3K1 depletion (Supplementary Figure 2A). Proliferation assays revealed that MAP3K1-deficient cell lines exhibited a mean ~2.4-fold increased proliferation rate and decreased sensitivity to both AZD8835 and AZD5363 (~2.61 and ~5.23-fold IC_50_ increases, respectively) compared with parental control cell lines (Supplementary Figure 2B). An example of a marked effect with AZD5363 is shown as a proliferation time course of CR2.5 cells compared to parental cells following treatment with either AZD8835 or AZD5363 (Figure 1C). Altogether these results indicated that MAP3K1 depletion in a *mPIK3CA* background decreased sensitivity to AZD8835 and promoted resistance to AZD5363. This was consistent with the hyper-activation of the PI3K pathway, exemplified by enhanced AKT phosphorylation and downstream signaling of pPRAS40, pP70S6K and pS6 RP.

### MAP3K1 depletion elicits stability of IRS1, impacting on activation of PI3Kα and Ras/Raf/Mek/Erk pathways

**Figure 2:**
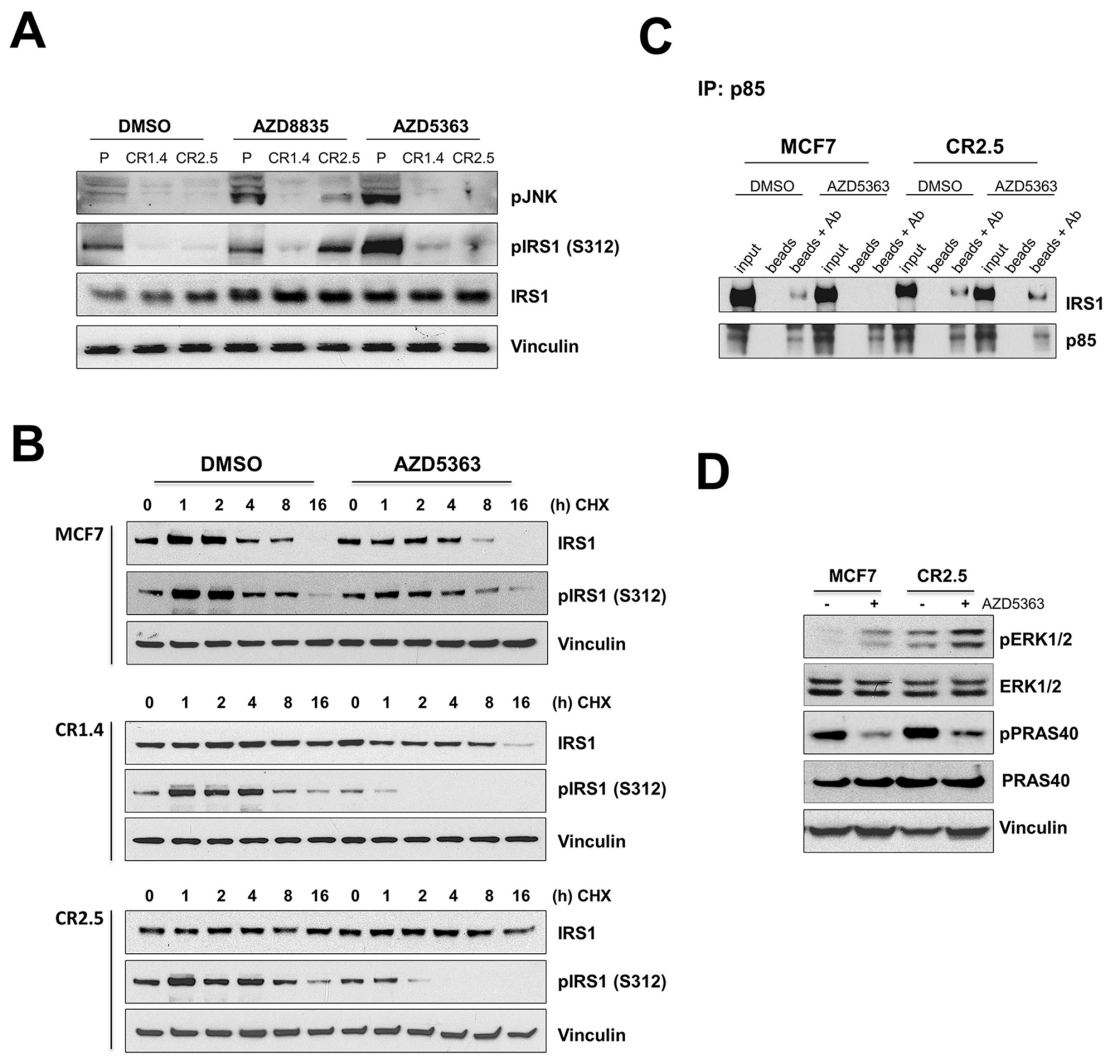
MAP3K1 depletion correlates with increased IRS1 stability **(A)** MCF7 parental, CR1.4 and CR2.5 cells were treated with 250 nM AZD8835, 250 nM AZD5363 or DMSO 24h prior to cell lysis. Cell lysates were collected and immunoblotted with indicated Abs. **(B)** MCF7 parental, CR1.4 and CR2.5 cells were incubated with 50μg/ml cycloheximide (CHX) at the indicated time points in the presence or absence of AZD5363 and immunoblotted with total- and phospho (Ser312)-IRS1 specific Abs. Densitometry quantification is shown in Supplementary Figure 2D. **(C)** Whole cell lysates were incubated with PI3K regulatory subunit (p85) Abs overnight. Immuno-complex pull-downs were performed using magnetic Protein A beads and immunoblotted with the indicated Abs. **(D)** Immunoblots from MCF7 parental and CR2.5 cells cultured in the presence (+) or absence (−) of AZD5363 and immunoblotted with the indicated Abs.

It has been reported that MAP3K1 downstream target, JNK, mediates IRS1 degradation *via* phosphorylation [22–24, 30]. We first aimed to confirm the effective inhibition of the MAP3K1-JNK signaling pathway in CRISPR modified MAP3K1-deficient cell lines by measuring pJNK levels (Figure 2A). pJNK levels were lower in MAP3K1-deficient cells compared with control parental MCF7 cells under normal conditions. In addition, as anticipated since JNK is a downstream target of the MAP3K1 pathway, MAP3K1-deficient cells were unable to activate JNK following AZD8835 and AZD5363 treatments, in contrast to parental cells (Figure 2A and Supplementary Figure 2C). Furthermore, as previously reported [22, 24], reduced activation levels in JNK correlated with a lower level of serine 312 phosphorylation on IRS1 (pIRS1 (S312)) (Figure 2A and Supplementary Figure 2C). To test whether altered IRS1 phospho-status might impact IRS1 stability we performed cycloheximide (CHX) pulse-chase experiments to determine IRS1 protein half-life (Figure 2B). IRS1 protein levels decreased after 4-8h CHX treatment and were undetectable at the 16h time point in parental control cells (Figure 2B). The kinetics in the MAP3K1 deficient cells changed significantly with stable IRS1 protein levels for at least 16h during CHX treatment. This observation was particularly remarkable in the MCF10A-PI3Kα^H1047R^ model, with undetectable IRS1 protein levels at any time point in control parental cells *versus* highly stable levels at 16h in the MAP3K1 deficient cells (Supplementary Figure 2D). In addition, we found that pIRS1 (S312), target of JNK [22, 23, 30] was differentially stimulated by AZD5363 in parental *versus* MAP3K1 deficient cell lines (Figure 2B and Supplementary Figure 2C). Hence we considered that the increase in IRS1 stability, associated with reduced pIRS1 (S312), could impact on overall IRS1 activity and PI3Kα output, as previously reported [31]. Therefore, we next examined whether this alteration in IRS1 stability could impact assembly of the PI3Kα complex [31] by immunoprecipitating proteins associated with the PI3Kα regulatory subunit p85 [32, 33]. We were able to immuno-precipitate IRS1 in DMSO treated MCF7 parental cells together with p85 but not after AZD5363 treatment (Figure 2C). In contrast, in MCF7 CR2.5 cells, IRS1 immuno-precipitated with p85 in both DMSO and AZD5363-treated cells (Figure 2C). Altogether, this may suggest that deficiency in MAP3K1-JNK pathway signaling results in decreased pIRS1 (S312) levels, thereby promoting IRS stability and maintained IRS1/p85 interaction, which is directly associated with PI3Kα activity. In particular, our findings suggest that at least part of the AZD5363 resistance mechanism in MAP3K1-deficient cells might be explained by resilient IRS1/p85 interaction. However, we were mindful that IRS1 facilitates the activation of at least two cell signaling pathways, PI3Kα and Ras/Raf/Mek/Erk [32, 34]. Also, despite partial PI3K pathway blockage following AZD5363 treatment (Figure 1B and Supplementary Figure 2A), MAP3K1-deficient cell lines seem able to sustain high proliferation rates (Figure 1C). Therefore, to further explore how MAP3K1 deficiency facilitates bypass of AZD5363-mediated PI3K pathway inhibition, we investigated whether ERK signaling might be additionally impacted by increased IRS1 activity, given a previous report that ERK1/2 can be stimulated, *via* Ras, following IRS1 activation [35]. We tested ERK1/2 activation levels in our model in basal conditions and following AZD5363 treatment (Figure 2D). We found that MAP3K1-deficient MCF7 cells had increased basal ERK1/2 activation level (phospho-ERK [pERK]) compared to parental MCF7 cells (Figure 2D). Further, AZD5363 treatment increased pERK levels in both control and MAP3K1-deficient cells, more prominently in the latter (Figure 2D), perhaps due to increased IRS1 stability. We therefore propose that ERK signaling acts as a possible escape route in MAP3K1-deficient cells, enabling bypass of AZD5363-mediated PI3K pathway blockage and sustaining cell proliferation.

### Organoid models reveal that MAP3K1 depletion promotes apoptosis resistance and increases proliferation

**Figure 3:**
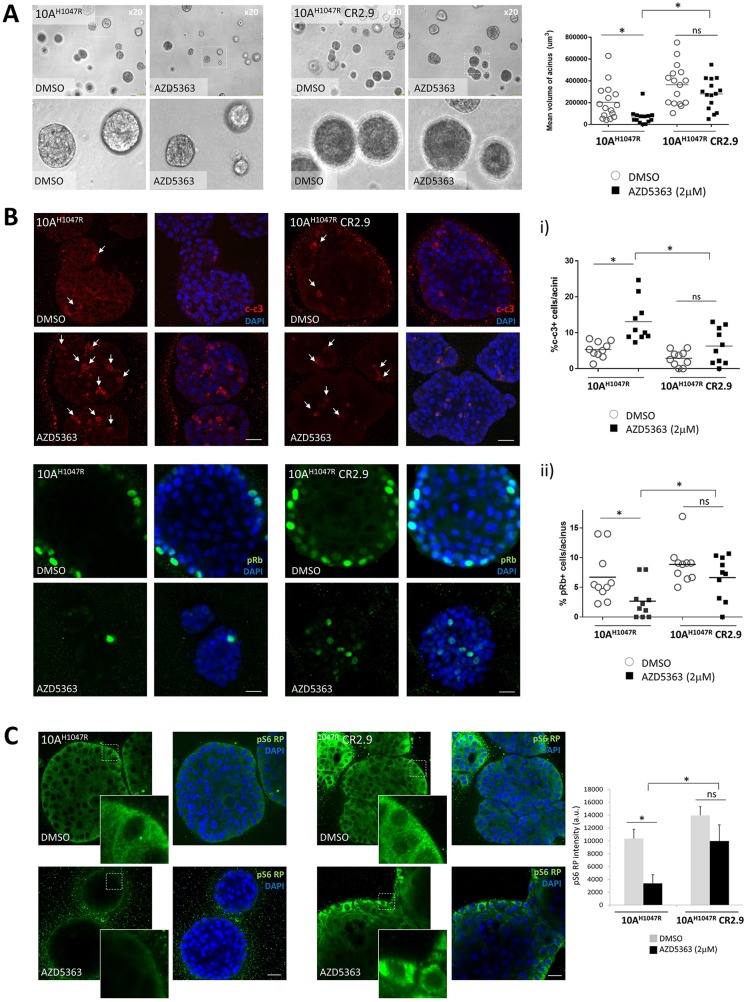
MAP3K1 depletion in 3D-MCF10A-PI3Kα^H1047R^ acini elicits apoptosis resistance and increased proliferation **(A)** Representative bright field images from parental and MAP3K1-deficient MCF10A-PI3Kα^H1047R^ acini atday 15 of morphogenesis. Acini were treated from day 4 to day 15 with 10 μM AZD5363 as indicated. Right graph represents mean volume of acini which was calculated using SPOT software following the equation [(length × width^2^)/2 = acinus volume (mm^3^)] (N = 10). **(B)** Confocal equatorial images from parental and MAP3K1-deficient MCF10A-PI3Kα^H1047R^ treated from day 4 to day 15 with 10mM AZD5363. At day 15 of morphogenesis acini were fixed and immunostained using cleaved caspase 3 (c-c3) (upper panels) and pRb (lower panels) Abs. Quantification of events is represented on right graphs (N = 20). **(C)** In identical conditions as in (b) acini were stained using pS6 RP Abs. Right graph shows the mean intensity in arbitrary units (a.u) of pS6 RP signal calculated with ImageJ (N = 20). Scale bars indicate 25 μm. P-values were determined by the Student's *t*-test.

In order to further characterize the effect of MAP3K1 depletion on proliferation and survival of *mPIK3CA* BrCa cells, we performed 3D-matrigel *in vitro* growth assays which represent a more physiological approach than regular 2D *in vitro* cultures [36–38]. In matrigel our MCF10A cells underwent a series of morphogenic events consistent with the development of acinar structures with a hollow lumen [36, 37, 39–42]. Measuring volume of 3D-acinar structures cultured in matrigel for 15 days, we observed that MAP3K1-deficient MCF10A-PI3Kα^H1047R^ acini (CR2.9) appeared larger compared with control MCF10A-PI3Kα^H1047R^ acini and their growth was significantly less inhibited by AZD5363 (Figure 3A). To determine the cause of increased acinus size in MAP3K1-deficient cultures we immuno-stained acini with apoptosis (cleaved caspase 3, c-c3) (Figure 3B, upper panels) and the proliferation (pRb) (Figure 3B, lower panels) markers. Luminal occlusion is a hallmark of early stages of breast tumorigenesis such as ductal carcinoma *in situ* [38, 43]. It has been shown that MCF10A acini stably expressing PI3Kα^H1047R^ exhibit luminal filling [44]. Accordingly, we observed this in images from equatorial sections of control DMSO-treated MCF10A-PI3Kα^H1047R^ and MCF10A-PI3Kα^H1047R^ MAP3K1-deficient (CR2.9) acini (Figure 3B). Apoptotic cells were primarily found in the lumen compartment and the number of events (~2 c-c3+/acinus) was similar both in control and CR2.9 acini (Figure 3B, graph i). As expected AZD5363 treatment increased significantly the number of apoptotic events (~12 c-c3+/acinus) in control MCF10A-PI3Kα^H1047R^ acini. However, AZD5363 treatment did not increase significantly the number of apoptotic events (~5 c-c3+/acinus) in MAP3K1-deficient acini (Figure 3B, graph i). Next, proliferative cells were stained for pRb in control DMSO-treated MCF10A-PI3Kα^H1047R^ and CR2.9 acini (Figure 3B, lower panels). In control conditions CR2.9 acini exhibited increased number of pRb positive cells (~8 pRb+/acinus) compared with parental MCF10A-PI3Kα^H1047R^ acini (~6 pRb+/acinus). Strikingly, whereas AZD5363 administration dramatically reduced the number of proliferative cells in parental MCF10A-PI3Kα^H1047R^ acini, it did not significantly reduce the number of pRb+ events in CR2.9 acini (Figure 3B, lower panels and graph ii). This suggests that MAP3K1 depletion enhances proliferation and promotes resistance to AZD5363-mediated induction of apoptosis and AZD5363-mediated inhibition of proliferation.

Next, we sought to understand whether the highly proliferative and low apoptotic phenotype observed in MAP3K1-deficient acini was accompanied by hyper-activation of the PI3K pathway and/or insensitivity to AZD5363 treatment. Previous 2D *in vitro* experiments had revealed that MAP3K1-deficient cell lines (MCF7 and MCF10A-PI3Kα^H1047R^ models) were resistant to AZD5363-mediated pS6 RP inhibition (e.g. Figure 1B). To test whether this effect prevailed also in 3D-acini we immuno-stained for pS6 RP in MCF10A-PI3Kα^H1047R^ acini (Figure 3C). Confocal equatorial sections revealed comparable levels of pS6 RP signal in both control and MAP3K1-deficient acini when treated with DMSO. Matrigel substrate enables integrin and receptor tyrosine kinase coupling which transduces proliferative signaling in the basal compartment [45, 46]. Consistent with this, we observed increased pS6 RP intensity in the basal compartment of outer rim cells (Figure 3C, detailed images). AZD5363 reduced substantially pS6 RP signal in control MCF10A-PI3Kα^H1047R^ acini when compared with DMSO-treated acini. In contrast, pS6 RP levels remained considerably higher both in DMSO and AZD5363-treated CR2.9 acini, consistent with our previous 2D observations (Figure 1B) that MAP3K1 depletion results in AZD5363 resistance.

### MAP3K1-deficient tumors are relatively resistant to AZD5363 treatment

**Figure 4:**
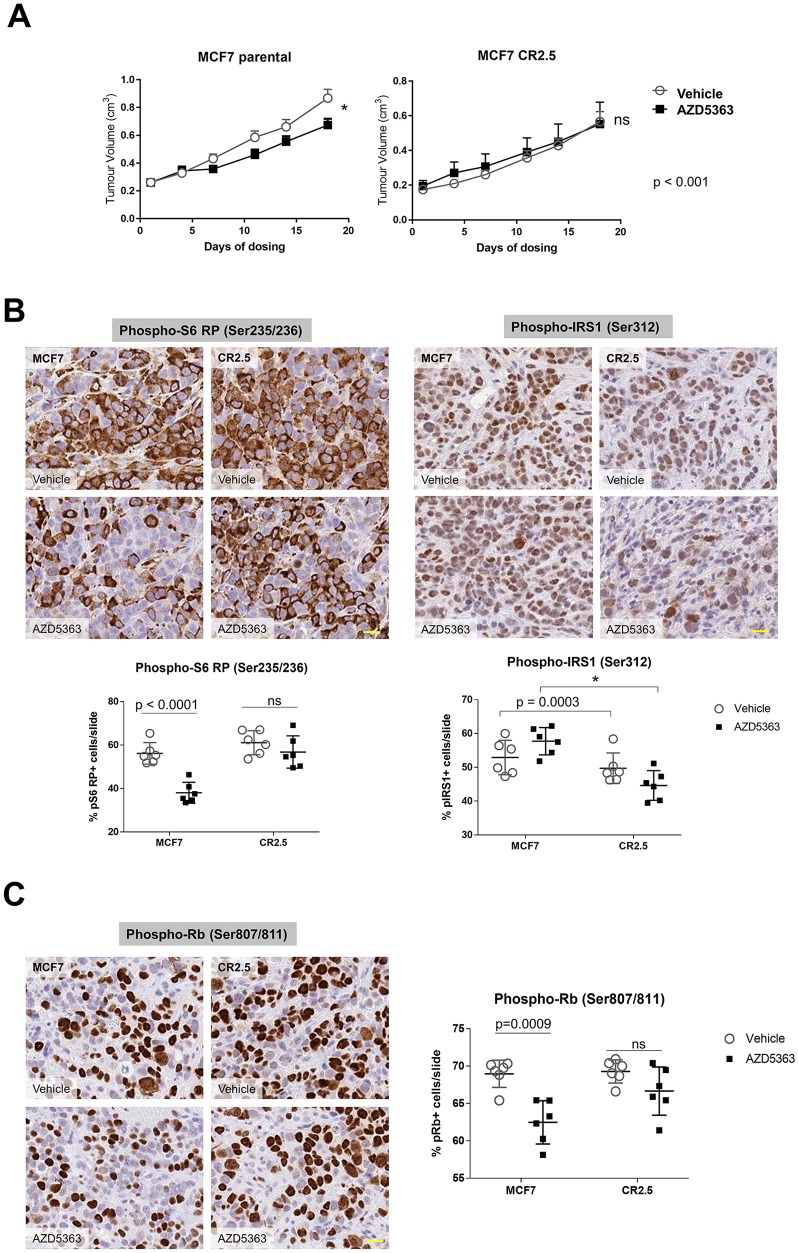
MAP3K1-deficient tumors are resistant to AZD5363 treatment **(A)** Quantification of tumor volume following vehicle or AZD5363 treatment in MCF7 parental (upper left panel) *versus* MCF7 CR2.5 (upper right panel) xenograft after 18 days dosing. P-values were determined by the Student's *t*-test. **(B** and **C)** Representative immunostainings of phospho-S6 RP, phospho-IRS1 (B) and phospho-Rb (C) markers in MCF7 parental and MCF7 CR2.5 xenografts sections from xenograft tumors. Scale bars (yellow) represent 20 μm. Graphs show analysis and quantification of positive events using HALO (indica labs). The resulting algorithm was applied to the whole slide (n = 6) (see Supplementary Figure 4 for more details). P-values were determined by the Student's *t*-test.

Our evidence from 2D and 3D *in vitro* studies supported the notion that MAP3K1 depletion elicited elevated proliferation rates and reduced sensitivity to AKT inhibition. We then sought to confirm our observations *in vivo* using a xenograft model. We implanted MCF7 parental and CR2.5 cell lines in mice and treated with 150 mg/kg BID AZD5363 for 28 days. We measured tumor volumes and observed that AZD5363 treatment significantly reduced (22%) mean tumor volume (MTV) of MCF7 parental tumors whereas it had no effect on MCF7 CR2.5 tumors, at day 18 of treatment (Figure 4A). Notably, we quantified a significant MTV reduction (27%) in MCF7 parental *versus* a non-significant MTV reduction (18%) in MCF7 CR2.5 tumors at day 28 of treatment (Supplementary Figure 3A). These data indicate that MCF7 CR2.5 xenografts exhibit reduced sensitivity to the AKTi, suggesting that MAP3K1 deficiency promotes resistance to AZD5363 *in vivo*.

Surprisingly, we observed that prior to dosing (day 18 post-implantation) MTV of MCF7 CR2.5 tumors was significantly smaller than parental tumors (Supplementary Figure 3B, left panel). Evidence in the literature indicates that an activated MAP3K1-JNK axis correlates with enhanced cell growth and size [47, 48] and we observed that regardless of background (cell area quantification of bright field images from MCF7, and MCF10A-PI3Kα^H1047R^ or T47D *in vitro* cultures [not shown]) MAP3K1-deficient cells appeared smaller than parental. Thus we hypothesized that the smaller size of CR2.5 tumors *versus* parental tumors could be attributable to the smaller size of CR2.5 mutant cells. To investigate this, we performed cell number count and cell area estimation using HALO software (Supplementary Figure 3B, middle and right panels). We demonstrated that CR2.5 tumors exhibited increased number of cells per area. Further, the size of these cells was significantly smaller than in parental MCF7 tumors.

To mechanistically characterize the function of MAP3K1 in this AZD5363-resistant phenotype we first sought to understand whether MAP3K1 depletion was impacting PI3K pathway signaling. On vehicle treatment, we observed a non-significant trend toward increased pS6 RP activity in MCF7 CR2.5 *versus* parental tumors (Figure 4B and Supplementary Figure 4A, left panels). In addition, while AZD5363 administration efficiently reduced pS6 RP activity in parental tumors, it had no effect on pS6 RP activity levels in CR2.5 tumors. This was in agreement with our previous *in vitro* studies (Figure 1B and 3C), indicating that MAP3K1-depletion correlates with increased PI3K pathway signaling and AZD5363 resistance. Next, we sought to understand whether MAP3K1-depletion has a functional effect on IRS1 activity *in vivo*. We immune-stained parental and CR2.5 tumors sections for pIRS1 (Ser312). We did not observe any effect either in parental and CR2.5 tumors in vehicle-treated conditions (Figure 4B and Supplementary Figure 4A, right panels). However, a significantly greater reduction in pIRS1 activation levels was observed on AZD5363 treatment when comparing MAP3K1-deficient *versus* parental tumors. Again this result agreed with previous *in vitro* observations (Figure 2A and 2B). To test whether the pS6 RP and pIRS1 phenotype of MAP3K1-deficient xenograft had an impact on proliferation we measured pRb *in situ* by using immunostaining (Figure 4C). We observed a significant reduction in the number of pRb+ events in parental MCF7 tumors following AZD5363 treatment whereas this effect was not observed in MCF7 CR2.5 tumors (Figure 4C and Supplementary Figure 4B). This result suggests that in the context of xenograft tumor growth, MAP3K1 depletion counteracts the anti-proliferative effect of AZD5363 in tumor growth.

To summarize, genetic inactivation of *MAP3K1* in the context of *mPIK3CA* resulted in reduced sensitivity to the AKTi AZD5363. Importantly, upon AZD5363 treatment, MAP3K1-deficient tumors exhibited increased proliferation rate and were pS6 RP^HIGH^ and pIRS1^LOW^ when compared with parental controls, in agreement with our cell culture and organoids results.

## DISCUSSION

Here we identify a previously unrecognized cross-talk between the PI3Kα and MAP3K1/JNK pathway and demonstrate its potential impact in cancer therapy resistance. Specifically, we demonstrate that depletion of *MAP3K1* gene expression in the context of *mPIK3CA* BrCa cell lines causes resistance to PI3K pathway inhibition by PI3K pathway inhibitors, manifested as resistance to proliferation inhibition *in vitro*. Our results indicate that depleted MAP3K1/JNK signaling correlates with decreased pIRS1, enhanced IRS1 half-life and persistent binding to the PI3K regulatory subunit, p85. Moreover, *in vivo* studies demonstrate that MAP3K1 deficiency in MCF7 xenografts elicits relative AKTi inhibitor resistance. Immuno-histological analysis revealed that MAP3K1-deficient tumors are pRB^HIGH^ and c-c3^LOW^ suggesting a higher proliferation rate. Strikingly, these tumors are also pS6 RP^HIGH^, in agreement with our *in vitro* and 3D-organoid evidence showing reduced AZD5363 mediated inhibition of the PI3K pathway in a MAP3K1-deficient background. Specifically, our work provides important insights into how the mutational status of *PIK3CA* and *MAP3K1* genes could predict response to AZD5363 and other PI3K pathway inhibitors.

Given the PI3K pathway is frequently deregulated in cancer, our study raises important questions on how patient stratification based on mutational status of key components of this and other pathways might be critical in order to achieve optimal clinical anti-tumor efficacy. Due to the relatively disappointing outcomes of clinical trials targeting *mPIK3CA* tumors with PI3Kα inhibitors, a number of studies are currently under investigation targeting other PI3K pathway mediators [49]. We sought to evaluate the efficacy of the AKTi AZD5363, a distal PI3K pathway inhibitor, and interrogated its efficacy in different mutational scenarios. Our results suggest that tumors with concomitant *PIK3CA* gain of function mutation and *MAP3K1* LoF mutation are less responsive to AKTi treatment, at least in monotherapy. Our initial studies with the PI3Kα/δ inhibitor, AZD8835, pointed to similar findings.

One of the aims of this work was to characterize cross-talk between the PI3Kα and the MAP3K1 pathways, specifically, how this impacts on resistance to PI3K pathway inhibitors. With the exception of the study by Park H.S. *et al* which describes the direct phosphorylation of MAP2K4 by AKT [21], there is no evidence in the literature of a direct cross-talk between PI3Kα and the MAP3K1 pathways. Our study delineates first clearly identified cross-talk between MAP3K1-JNK and PI3K pathways, with IRS1 being a key nodal point connecting the pathways. Phospho-status of IRS1 determines its stability and subsequent PI3Kα kinase activity [50]. Further, post-translational modifications dictate its conformational structure that ultimately determine its affinity for the PI3Kα subunit, p85 [51, 52]. Stabilization of IRS1 in the PI3Kα complex correlates with signaling and this appears critical in explaining the resistance mechanism to AZD5363.

We observed that AZD5363 and AZD8835 treatment increases MAP3K1 expression (Supplementary Figure 1E); this increase correlates with enhanced phospho-JNK activity (Figure 2A) suggesting full activation of the pathway. We have not investigated the mechanisms underlying this increase in pJNK but we believe this could be anticipated since MAP3K1 is a recognized sensor of external insults and it is reasonable that MAP3K1 could be induced following administration of therapeutic agents [12]. This mechanism might have positive anti-tumor effects, since MAP3K1 induction following AZD5363 and AZD8835 administration would elicit (*via* JNK) IRS1 Ser312 phosphorylation and IRS1 degradation and thereby oppose counteracting signals in response to PI3K pathway inhibition such as from the p70^S6K^ feed-back loop [25].

**Figure 5:**
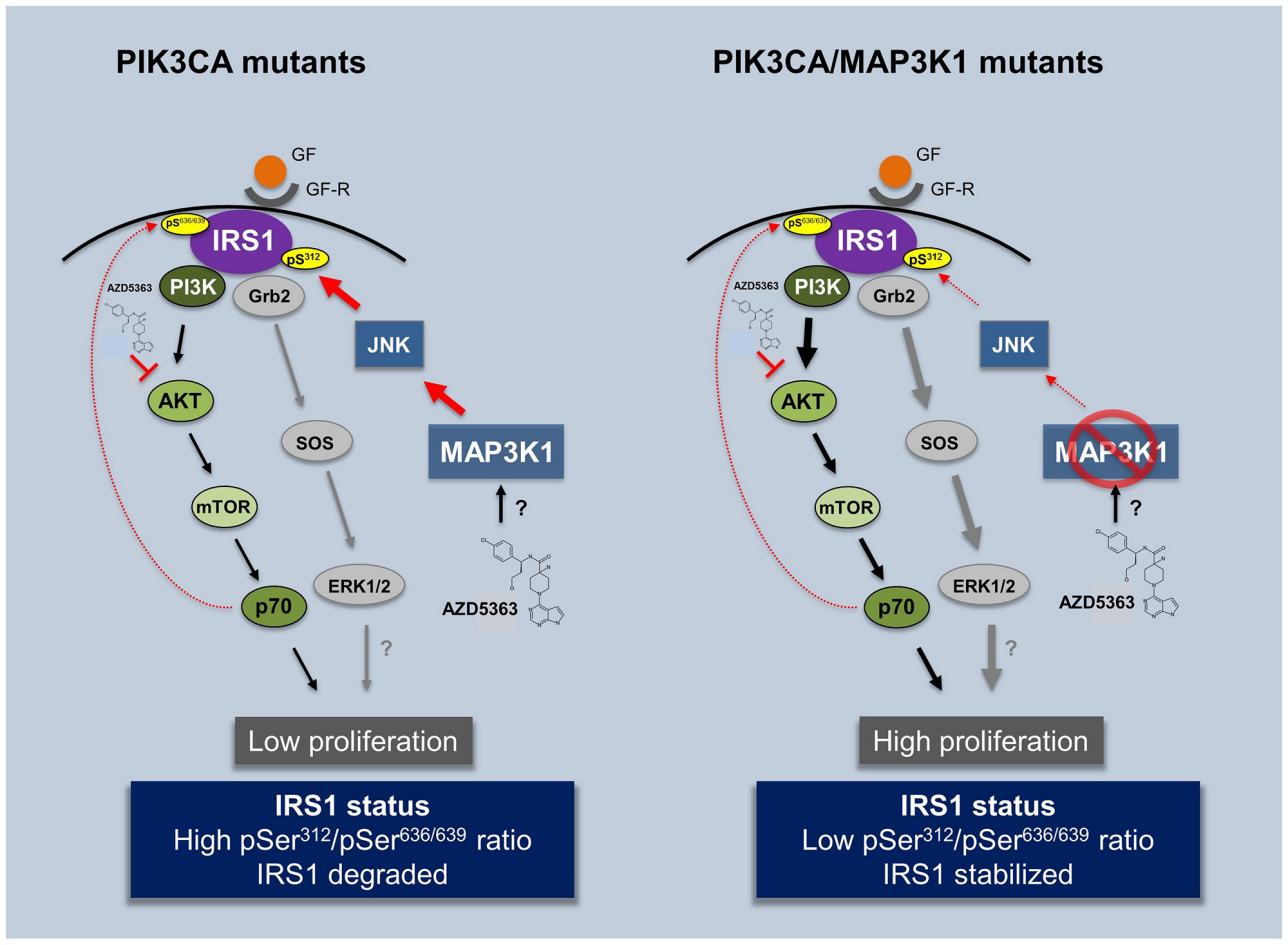
Impact of AZD5363 treatment in *PIK3CA* and double mutant *PIK3CA/MAP3K1* patients Illustration contemplating experimental (*in vitro*, 3D organoids and *in vivo*) and clinical observations presented in the study. AZD5363 can be used as an efficient compound to treat *mPIK3CA* tumors by blocking the pathway at the level of AKT. However its efficiency may be compromised in patients harboring co-occurring *MAP3K1* LoF mutation. Patients with reduced MAP3K1 expression have deregulated JNK pathway activity and consequently decreased phospho-serine IRS1 (S312) levels. This increases IRS1 stability and subsequently brings about the re-activation of PI3Kα. pS, phospho-serine, GF, growth factor, GF-R, growth factor receptor.

In the present study, MAP3K1-deficient cells exhibit a significant increase of PI3K pathway activation levels when compared with parental cells, even in the presence of AZD5363. Activation of AKT and downstream targets leads to inhibition of apoptosis, cell survival and correlates with tumor progression [53]. Although this may in part explain the AZD5363-resistant phenotype of MAP3K1-deficient tumors, we suspected that other pathways may be involved in bypass of AKT blockage. Since IRS1 also mediates Ras activation, one obvious candidate was the Ras/Raf/Mek/Erk pathway [54, 55]. In our hands, AZD5363 administration correlates with high pERK levels and this is further enhanced when MAP3K1 is depleted (Figure 2D and Figure 5, right scheme). We also propose that increased IRS1 stability as a consequence of MAP3K1-depletion results in enhanced Ras/Raf/Mek/Erk signaling. We propose that drugs targeting PI3Kα signaling are sub-optimally effective because their effects are dampened by activation of parallel pathways such as Ras/Raf/Mek/Erk. On the contrary, co-administration of AKT with MEK inhibitors [56] can attenuate the negative feedback, restore AKTi sensitivity and may ultimately provide therapeutic benefit for patients with concomitant *PIK3CA/MAP3K1* mutations.

Although some previous investigations clearly point toward a tumor suppressor role of MAP3K1 in the context of *mPIK3CA*, overall bibliographic evidence is not conclusive [4, 6–11]. Tumor micro-environment is populated with a repertoire of different cellular subtypes that are implicated in numerous cellular responses (i.e. angiogenesis, immune response and metabolism) that ultimately impact on tumor fate and development [57, 58]. Therefore to further characterize the phenotypic impact of MAP3K1 depletion in tumor fate we implanted parental and MAP3K1-deficient MCF7 cells in nude mice and followed their growth for 28 days. Significant differences were observed when tumors were challenged with AZD5363; MAP3K1-deficient tumors were less sensitive to AZD5363, displaying higher proliferation rates compared with parental counterparts. Importantly, whereas AZD5363 efficiently reduced pS6 RP levels in parental tumors, these levels remained unchanged in MAP3K1-deficient ones, consistent with resistance to AKT inhibition.

We have observed that MAP3K1 is induced in the presence of PI3K pathway inhibitors such as AZD5363 or AZD8835 (Supplementary Figure 1E), in agreement with MAP3K1's role as transducer of external stress insults. We hypothesize that in the m*PIK3CA* context, AZD5363 exposure triggers enhanced MAP3K1 levels and high pIRS1 (Ser312) activity. This negatively impacts IRS1 stability resulting in reduced recruitment of the PI3Kα regulatory subunit (p85) and reduced PI3K pathway signaling (Figure 5, left scheme). Thus, in this scenario AZD5363 has two additive anti-proliferative effects, firstly by blocking AKT directly and secondly via stimulating MAP3K1 which provides negative feed-back signaling through the IRS1-PI3Kα axis. By contrast in the *PIK3CA/MAP3K1* dual mutation scenario AZD5363 is less effective (Figure 5, right scheme), due to hyperactivation of the PI3K pathway *via* IRS1; furthermore, the loss of MAP3K1-JNK signaling allows for PI3K pathway bypass through deregulation of additional oncogenic pathways, for example Ras/Raf/Mek/Erk pathway [32, 34]. Preliminary observations indicate that ERK activity is enhanced in the MAP3K1-deficient context and is further enhanced in the presence of AZD5363. Although more insights need to be accrued, we propose the Ras/Raf/Mek/Erk pathway is a potential route by which AKT inhibition can be bypassed.

In conclusion, our investigations indicate that *MAP3K1* LoF mutations, in the context of a *mPIK3CA* background, may be considered as predictive biomarkers to evaluate efficacy of PI3K pathway inhibitors.

## MATERIALS AND METHODS

### *In vivo* studies

MCF7 parental and MAP3K1 deficient (CR2.5) cell lines were implanted subcutaneously in the flank of male SCID mice with estrogen pellet (0.5mg/21 days). Cells were implanted at a concentration of 5 × 10^6^ cells/mouse with Matrigel at a total volume of 0.1mL. When tumors reached approximately 0.2 cm^3^ they were randomized into two groups, vehicle (10% DMSO, 25% Kleptose) and AZD5363 treatment (150 mg/kg) BID (twice daily), for 28 days. Mice were treated 32 times following a schedule (4 days on, 3 days off for 3 cycles and on the 4th cycle tumors were dosed 4 days on) and subsequently sampled. Two hours post morning dose on the day of the last compound administration, tumors were excised and collected for analysis. All procedures were carried out in accordance with UK home office regulations and with approved institutional guidelines.

### Cell lines and treatments

Low-passage MCF10A-PI3Kα^H1047R^ (from Horizon Discovery, Cambridge, UK) cells were cultured in the same conditions as parental MCF10A cells as described previously [40, 42]. MCF7 cells where cultured in phenol-free RPMI medium reconstituted with 10% fetal calf serum. For AZD5363, AZD8835 and cycloheximide experiments cells were plated a day prior to treatment and drugs added in fresh medium at the indicated times and concentrations. For 3D cultures, cells were plated in growth factor reduced (GFR) BD Matrigel basement membrane matrix, phenol red free (BD Biosciences, San Diego, CA, USA) and grown as described [38]. For the proliferation assays cells were plated in 96-well plates at a 2000 cell/well density and confluence was measured with IncuCyte technology (Essen Bioscience, Ann Arbor, MI, USA).

### Western blots

MCF10A and MCF7 cells were lysed in AZ cell panel lysis buffer supplemented with protease inhibitor cocktail (Roche, Welwyn Garden City, UK) and phosphatase inhibitor (Thermo Fisher Scientific, MA, US). Protein concentration was quantified with Pierce™ BCA Protein Assay Kit (Thermo Fisher Scientific) and equal amounts of protein were loaded and separated in Mini-PROTEAN® precast gels (Bio-Rad, CA, US). Membranes were transferred with iBlot^®^ 2 Transfer Stacks (Thermo Fisher Scientific, MA, US) and blotted against the following antibodies: phospho-SAPK/JNK (Thr183/Tyr185), phospho-Rb (Ser807/811), phospho-S6 RP (Ser253/225), phospho-AKT (Thr308), phospho-PRAS40 (Thr246), cleaved caspase-3 (Asp175), phospho-ERK1/2 MAPK (Thr202/Tyr204), AKT, IRS1, phospho-IRS1 (Ser312), phospho-p70 S6 kinase (Thr421/Ser424), all purchased from Cell Signaling Technology (CST, MA, US); MAP3K1 (Santa Cruz Biotechnology, TX, US) and Vinculin (Sigma-Aldrich, MO, US). Anti-mouse or anti-mouse IgG horseradish peroxidase-linked secondary antibodies (CST) and Supersignal (Thermo Fisher Scientific) were used to detect bound antibodies.

### Immuno-precipitation

Cell lysates were quantified and equal amounts were subject to immunoprecipitation overnight with PI3 Kinase p85 antibody (CST) or IgG mouse (CST) isotype control and Dynabeads Protein G (Invitrogen). Immuno-complexes where washed thrice with AZ cell lysis panel buffer and resuspended in pure water and NuPAGE LDS sample buffer and sample reducing agent (Thermo Fisher Scientific).

### CRISPR gene editing

#### Selection of target sites

Two CRISPR target sites (Cr1: 5′-CAAGATGGATGATCGTCCAG-3′ and Cr2: 5′-AGCCTGGAAGCACGAATGGT-3′) against the second exon of the human *MAP3K1* gene were selected using MIT's CRISPR design tool (http://crispr.mit.edu/). Guide-RNAs (gRNAs) expression plasmids were constructed by ligating oligonucleotide duplexes into an AarI-linearized plasmid containing human U6 promoter and an optimized coding sequence of wild-type SpCas9 (Streptococcus pyogenes Cas9) under the control of CMV promoter. The sequence of the recombinant plasmids was confirmed using Sanger sequencing.

#### Surveyor assay

The mutant clones at MAP3K1 target sites were identified using Surveyor assay. Briefly, on- and off-target sites were amplified from 50 ng of genomic DNA using Fusion-Flash HiFi DNA polymerase (Life Technologies) and primers (Supplementary Table 1). 10 μl of PCR products were denatured and reannealed using a thermocycler with the following protocol: 95°C, 10 min; 85°C, 1 min; 75°C, 1 min; 65°C, 1 min; 55°C, 1 min; 45°C, 1 min; 35°C, 1 min; 25°C, 1 min (the temperature ramping between each step was set at −1°C/sec); hold at 4°C. Hybridized PCR products were treated with 1 μl of Enhancer and 1 μl of Surveyor endonuclease (IDT) at 42°C, 40 min. Reactions were stopped by the addition of 1.5 μl of the Stop solution (provided by the Surveyor kit) and digestion products were visualized on 10% TBE Acrylamide gel. Mutations at target sites were then confirmed using Sanger sequencing and deep targeted next generation sequencing.

#### Sanger sequencing of mutant alleles

The target region of *MAP3K1* gene from each clone was amplified and purified using QIAGEN PCR purification kit following the manufacturer's instruction. Purified fragments were then A-tailed using Ampli-Taq DNA polymerase kit (Roche) at 72°C, 30 min and were cloned into PCR2.1 TOPO vector using a Topo cloning kit and transformed into One-Shot E. coli competent cells (Life Technologies). Plasmid DNA was isolated for at least 5 colonies from each transformation and then sequenced using M13Fwd primer.

#### Targeted deep next generation sequencing (NGS)

The *MAP3K1* knockout cells were further confirmed using NGS. Briefly, the targeted *MAP3K1* site was amplified using Fusion-Flash HiFi DNA polymerase (Life Technologies) using the primers listed in Supplementary Table 1. PCR products were generated from 50ng of genomic DNA extracted from wild-type and knockout cells. Amplified PCR products were subjected to paired-end sequencing using a NextSeq500 NGS instrument (Illumina). Finally, sequencing reads were automatically de-multiplexed using NextSeq500 Instrument and paired FASTQ files were analyzed using CRISPResso [44]. The frequency of mutated alleles among NGS reads were calculated based on the CRISPResso's detected alleles (Supplementary Figure 1).

### siRNA targeting

Cells were transiently transfected using Lipofectamine RNAiMax (Invitrogen, Grand Island, NY, USA). siRNA SMARTpool from Dharmacon (Lafayette, CO, USA) was purchased for targeting *MAP3K1* while a silencer negative control from Ambion (Grand Island, NY, USA) was used as a negative control. Transfections of siRNA *MAP3K1* oligonucleotides were performed in six-well plates at a final concentration of 10 nM siRNA oligonucleotides and incubated for 48 hours according to the manufacturer's instructions.

### Confocal microscopy

Alexa Fluor 488 or 594 conjugated secondary antibodies (Thermo Fisher Scientific, MA, US) were used for 3D culture immunofluorescence assays. MCF10A-PI3Kα^H1047R^ 3D-acini were fixed (4% PFA) at day 15 and processed for immunofluorescence microscopy analysis as established previously [59]. Confocal analyses were performed using the Leica SP5 Multi-photon confocal microscope equipped with UV diode (405 nm), argon (458, 476,488 and 514 nm), HeNe (543 nm) and a HeNe (633 nm) lasers. All images were obtained with a x63 objective. Quantitative measurements of optical density were performed with ImageJ (National Institutes of Health, US). Acinar size was calculated with LAS X software (Leica) following the equation [(length × width^2^)/2 = acini volume (mm^3^)] and plotted with GraphPad Prism.

### Immuno-histochemistry

The immunostaining protocol was performed as described previously [60]. In summary, sections were dewaxed in 2 changes of xylene, 2 changes of absolute alcohol and 1 change of 70% alcohol. Antigen retrieval was performed in pH 6 buffer and heated to pressure at 110°C, for 2 minutes. Slides were subsequently processed on the Labvision Autostainer for peroxidise blocking (3%), serum block (Dako X0909), primary antibody incubation (cleaved caspase 3 diluted 1:250 and p-Rb, p-S6 RP and p-IRS1 diluted 1:200), secondary Ab incubation (Dako Envision+/HRP) and final liquid DAB+ (Dako K3468) incubation.

### Statistics

Statistical analyses were performed using GraphPad Prism 5.0 software (GraphPad software) and P-values were calculated using one-way analysis of variance followed by the Bonferroni multiple comparison post-test or the unpaired Student's *t*-test with P<0.05 considered statistically significant; N.S., not significant. Mann-whitney u test was used for the non-normal distributions (*in vivo* data).

## SUPPLEMENTARY MATERIALS FIGURES AND TABLES


