# Supplementary material for: Functional significance of co-occurring mutations in PIK3CA and MAP3K1 in breast cancer
Source: Oncotarget. 2018 Apr 20;9(30):21444–58. doi: 10.18632/oncotarget.25118 (PMC5940413; doi:10.18632/oncotarget.25118)
Supplement: Supplementary file 1 [file oncotarget-09-21444-s001.pdf]

Functional significance of co-occurring mutations in *PIK3CA* and *MAP3K1* in breast cancer

SUPPLEMENTARY MATERIALS

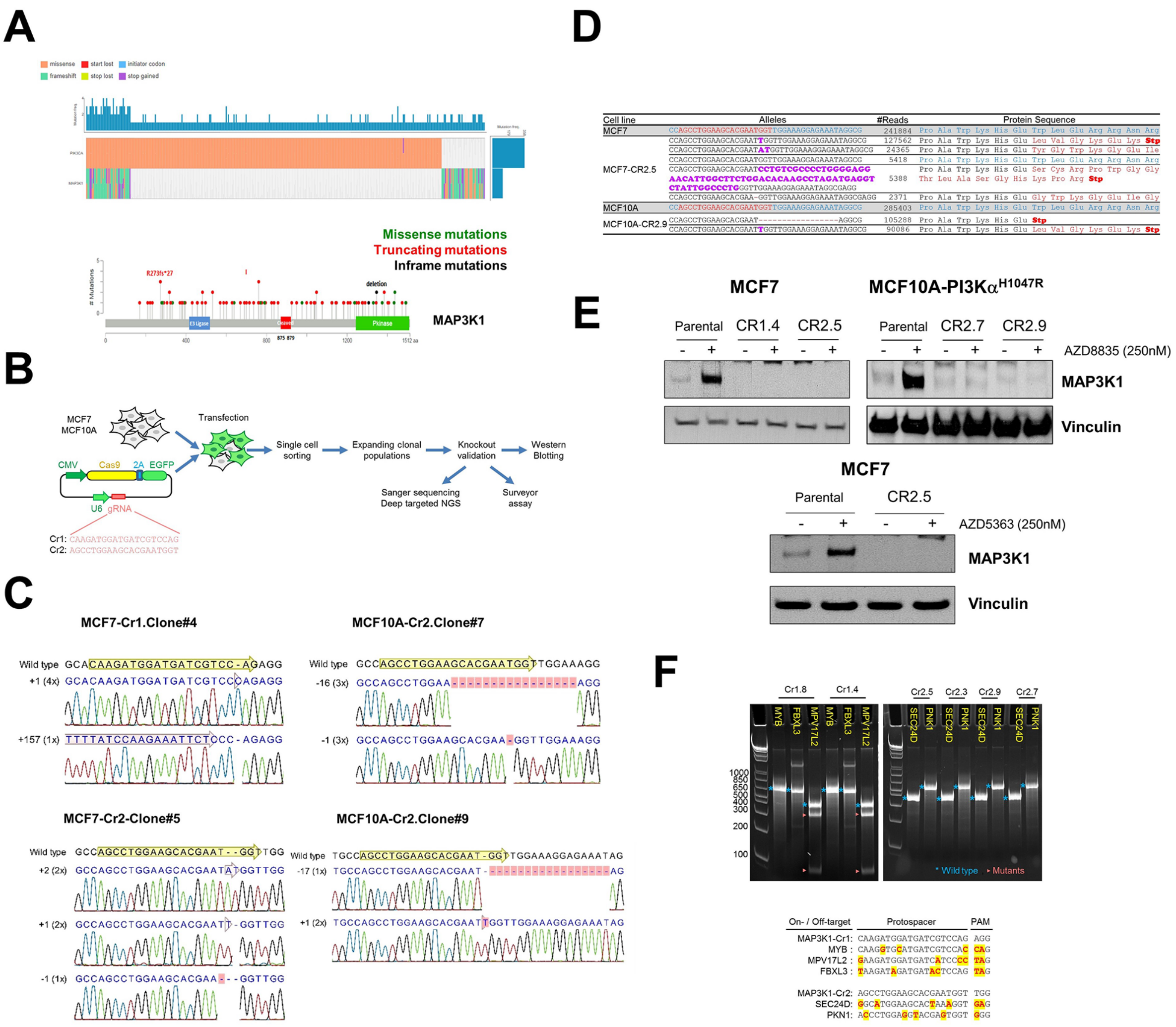

Supplementary Figure 1: (A) Schematic diagram representing the *MAP3K1* and *PIK3CA* mutations in a subset of ER+ samples (356 patients; TCGA database). Patients harboring co-occurring *MAP3K1* LoF mutations and *PIK3CA* activating mutations are clustered in the left side of the graph. The lower graph illustrates the distribution of mutations across MAP3K1 functional domains, where green, red and black represent missense, truncating and in-frame mutations, respectively. **(B)** Schematic diagram showing the overall experimental approach for generating and validation of MAP3K1-/- cell lines. The Cas9 expression plasmids use the CMV promoter for transient expression of Cas9 and human U6 promoter for expressing the gRNA in order to mediate a double strand break at targeted sites. EGFP, enhanced green fluorescent protein; 2A, self-cleavage peptide. **(C)** Mutated *MAP3K1* alleles from gene-modified clones were discriminated using Sanger sequencing. The wild-type sequence is shown at the top of the alignments with both target sites highlighted in yellow. Alleles detected multiple times are indicated with the numbers in parenthesis. Detected alleles were inactivated thorough the interruption and subsequent creation of frameshifting mutations within the coding sequence of *MAP3K1*. Deletions are indicated by dashes against a light red background. **(D)** NGS results for two of the knockout cell lines derived from MCF7 and MCF10A cells confirmed more than 95% of the alleles were out-of-frame mutations. **(E)** Upper panel, parental MCF10A-PI3Kα^{H1047R} and MAP3K1 deficient cells (CR2.7 and CR2.9) were cultured for 48h and treated with DMSO (-) or 250 nM AZD8835 (+) 24h prior to lysis. Lower panels, parental MCF7 and *MAP3K1*-deficient cells were cultured for 48h and treated with DMSO (-), 250 nM AZD8835 (CR1.4, CR2.5) or 250 nM AZD5363 (CR2.5) (+) 24h prior to lysis. Cell lysates were immunoblotted with the antibodies indicated. **(F)** Analysis of off-target sites in knockout cell lines is shown. Mismatches at off-target sites are highlighted in yellow. We did not detect off-target mutations in either MCF7 CR2.5 or MCF10A-PI3Kα^{H1047R} CR2.9 cell lines.

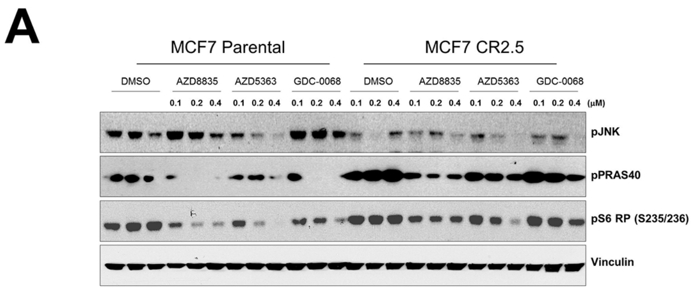

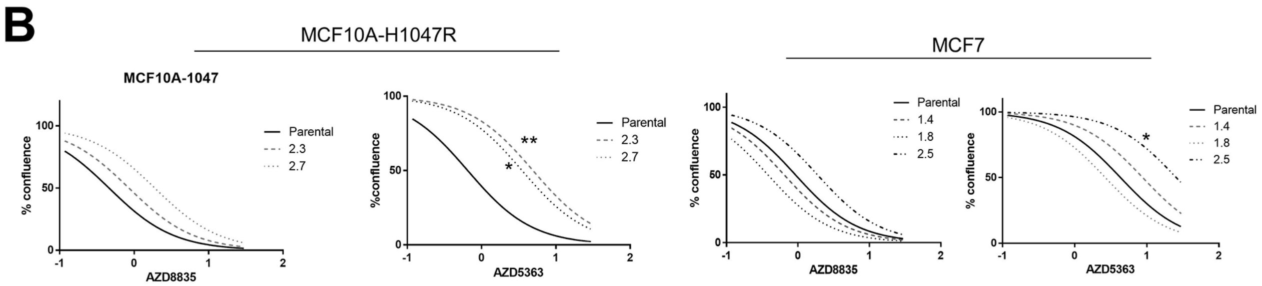

	H1047R	CR2.3	CR2.7
AZD8835 (μM)	0.5370	1.179	2.269
AZD5363 (μM)	0.4240	5.0395	4.916

	MCF-7	CR1.4	CR1.8	CR2.5
AZD8835 (μM)	0.9255	0.6436	0.3809	1.883
AZD5363 (μM)	4.314	8.772	2.656	25.6

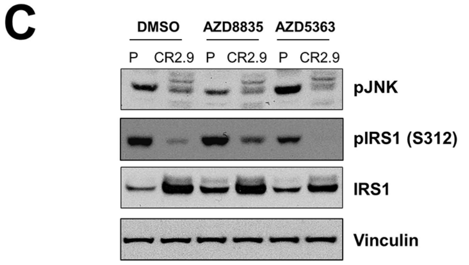

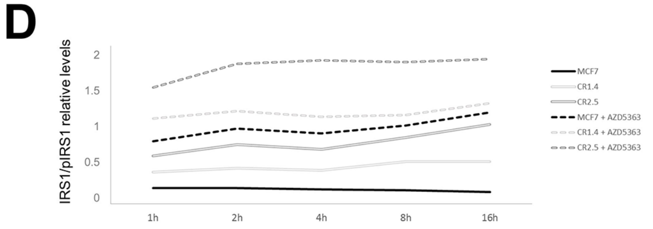

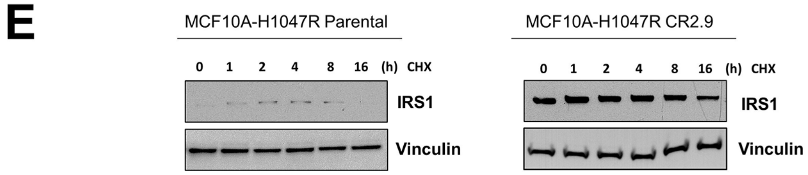

Supplementary Figure 2: (A) MCF7 parental and CR2.5 *MAP3K1*-deficient cells treated 24h prior to lysis with DMSO, AZD8835, AZD5363 or GDC-0068 at the indicated concentrations. Cell lysates were then immunoblotted with indicated antibodies (Abs). **(B)** Comparative proliferation curves of parental MCF7 or MCF10A-PI3Kα^{H1047R} *versus* MCF7 (CR1.4, CR1.8, CR2.5) or *MAP3K1*-deficient MCF10A-PI3Kα^{H1047R} (CR2.3, CR2.7) cultures, respectively, analyzed using IncuCyte. For IC$_{50}$ calculation (lower tables) cultures were grown in increasing concentrations (117 nM to 30 μM) of AZD8835 (left panels) or AZD5363 (right panels) for 72h and normalized against DMSO controls. Significance was determined using One-way ANOVA test with * $p < 0.05$. Underlined in the table are mutant cell lines with IC$_{50}$ curve significantly different from parental control cultures. **(C)** Parental MCF10A-PI3Kα^{H1047R} and *MAP3K1*-deficient cells (CR2.9) were cultured for 48h and treated with DMSO, 250 nM AZD8835 or 250 nM AZD53635 24h prior to lysis. Cell lysates were collected and immunoblotted with indicated Abs. **(D)** IRS1/pIRS1 ratios from Figure 2B immunoblots were quantified using ImageJ Software. **(E)** Parental MCF10A-PI3Kα^{H1047R} and *MAP3K1*-deficient cells (CR2.9) were incubated with 50 μg/ml cycloheximide (CHX) at the indicated time points and immunoblotted with total IRS1-specific Ab.

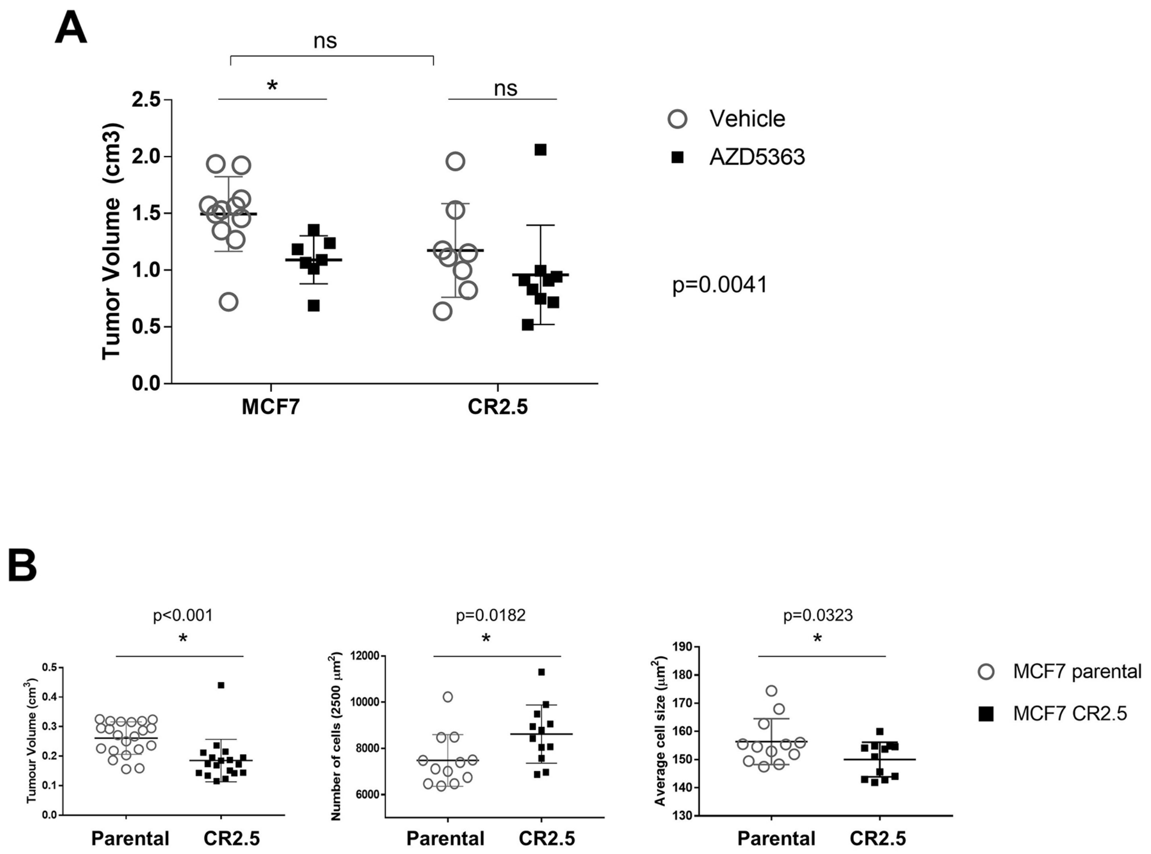

Supplementary Figure 3: **(A)** Quantification of tumor volume following vehicle (blue) or AZD5363 (red) treatment in MCF7 parental *versus* MCF7 CR2.5 xenograft at end of study (day 28). **(B)** Graphs showing mean tumor volume (left), number of cells per area (middle) and average cell size (right) in parental MCF7 *versus* CR2.5 xenografts. On the left graph each dot represents a mouse tumor measurement prior to randomization and dosing. Middle graph, using HALO the total number of cells was estimated in 5 different areas (500 μm^2 each) of a tumor H&E section (N = 12). Right graph, using HALO average cell size was estimated in an entire H&E slide (N = 12). P-values were determined by the Mann Whitney test.

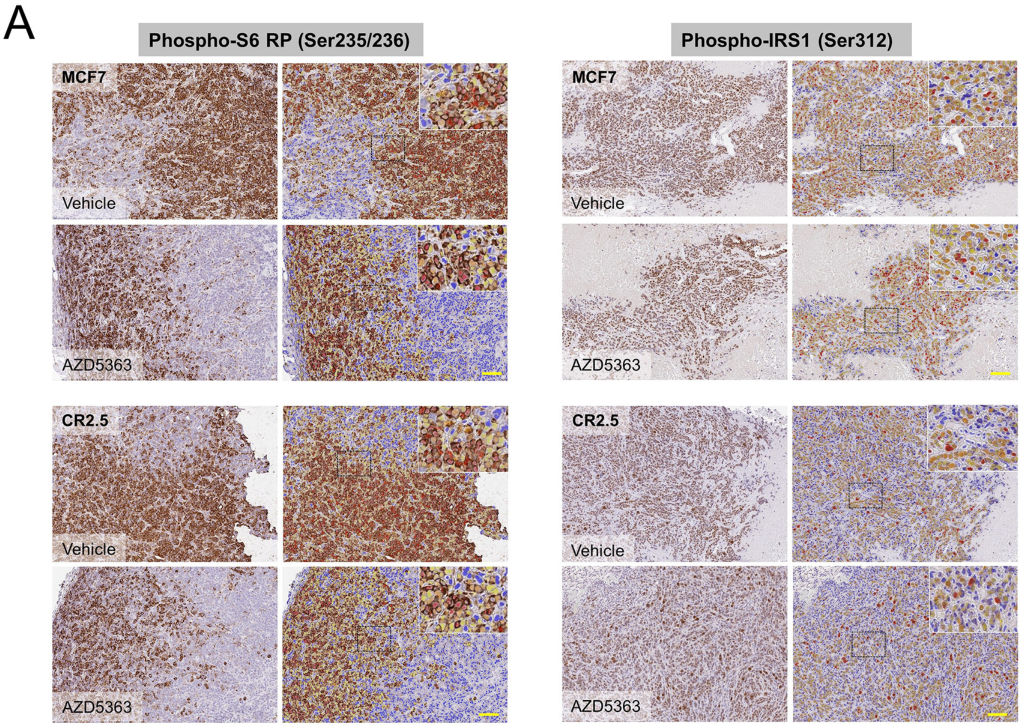

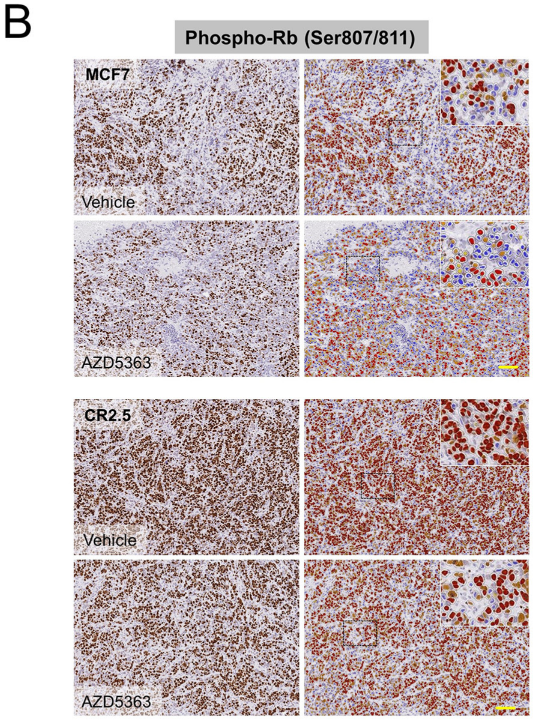

Supplementary Figure 4: **(A and B)** Representative immunostainings from xenograft tumors of the indicated biomarker endpoints. Images on the right of every set shows the algorithm designed for quantification using HALO (Indica labs). Insets are magnifications of the dotted black square box. Scale bars (yellow) represent 100 μm.

Supplementary Table 1: List of oligonucleotides used in this study

Oligo name	Sequence (5′-3’)	Application
Cr1-OligoTop	ACCGCAAGATGGATGATCGTCCAG	Guide RNA cloning
Cr1-OligoButtom	AAACCTGGACGATCATCCATCTTG	Guide RNA cloning
Cr2- OligoTop	ACCGAGCCTGGAAGCACGAATGGT	Guide RNA cloning
Cr2- OligoBottom	AAACACCATTCGTGCTTCCAGGCT	Guide RNA cloning
MAP3K1-Fwd	AGTCATTTGGGGTTTTTAGCAGT	Surveyor and Sanger
MAP3K1-Rev	TGAGAGGGCAGTCTTGGTTT	Surveyor and Sanger
MAP3K1-NGS-Fwd	*TCGTCGGCAGCGTCAGATGTGTATAAGAGACAG*<br>TGCCTGCATTTTAGTCGTGAG	Deep targeted NGS
MAP3K1-NGS-Rev	*GTCTCGTGGGCTCGGAGATGTGTATAAGAGACAG*<br>CCCATAGCCACTTACCACAGG	Deep targeted NGS
MYB-Fwd	CACAGAACCACACATGCAGC	Off-target analysis
MYB-Rev	TCAAACACCATTCCGTTCCCA	Off-target analysis
MPV17L2-Fwd	ATGCAAGACTGTCTCCTGGC	Off-target analysis
MPV17L2-Rev	GCCTGGCCCTATGTGAAACT	Off-target analysis
FBXL3-Fwd	ACAGAGTGAAGGGATGCCAC	Off-target analysis
FBXL3-Rev	AGGCTGACCTCATACTGTCCT	Off-target analysis
SEC24D-Fwd	GTGCCAAGCCACGCTAAGATA	Off-target analysis
SEC24D-Rev	GCCTCTAGTGACCCACGTTT	Off-target analysis
PKN1-Fwd	ATCTACCCCTCCAGACCAGC	Off-target analysis
PKN1-Rev	TCCGCTTCGGCTGTAAAGG	Off-target analysis
